# Local characterization of the COVID-19 response: the case of a lockdown in Lusaka, Zambia

**DOI:** 10.1186/s41256-021-00220-4

**Published:** 2021-09-30

**Authors:** Choolwe Muzyamba

**Affiliations:** 1 University College Utrecht, Campusplein 1, 3584 ED Utrecht, The Netherlands; 2 Present Address: Lusaka, Zambia

**Keywords:** COVID-19, Lockdown, Zambia

## Abstract

**Background:**

The onset of the COVID-19 pandemic has sparked heated debate among scholars on the relevance of lockdowns. There are those in favor of the lockdown and others who are critical of it. However, despite the increased interest in understanding the relevance of lockdowns, there still has not been much focus on its relevance in countries like Zambia. Thus, with the help of the Social Representation Theory (SRT), we set out to explore and document the local characterization of the lockdown by residents of Lusaka, Zambia.

**Methods:**

We recruited our participants through convenient and purposive sampling techniques. This was done through the use of the ZAMTEL public phone records. Initial contact was made to potential participants, and they were asked of their availability and willingness to participate in the interview. Upon agreeing to participate, they were included in the sample. A total of 68 people were selected to take part in this study. Their age ranged from 20 to 76 years old. 33 of them were male and 35 females. After this, we conducted interviews with the 68 participants. Due to COVID-19 restrictions, our interviews were conducted via telephone in conformity with the recommendations from the IRB in Lusaka and the advice of the ministry of health. We anonymized the demographic characteristics and responses from our participants. Later, thematic analysis was used to analyze the data.

**Results:**

The lockdown was on one hand lauded for slowing down the incidence rates, preventing fatalities, and protecting the healthcare system from collapse. On the other hand, it was criticized for exacerbating poverty levels, unemployment rates, increasing the rate of mental health problems, aiding gender-based violence, and intensifying political repression and corruption. The results speak to the complexity in the characterization of the lockdown as a response to COVID-19 in Lusaka, Zambia. This observation demonstrates the folly of viewing, applying and characterizing the COVID-19 lockdown as a ‘one-size-fits-all’ approach in Lusaka, Zambia.

**Conclusion:**

Rather than establishing the lockdown as an incontestable good, as it is depicted by some scholars or as useless by its critics, our findings instead demonstrate the diversity and complexity in how it is locally viewed by Lusaka residents. The study provides grounds for caution on simplistic and binary characterization of lockdowns. It indicates the need for careful dialog between the designers of lockdowns and citizens in order to tailor such interventions to local realities in context-specific ways. It also shows that though the development of such interventions, all the various and complex elements it embodies must be taken into account in order to realize optimum outcomes.

## Background

On March 2020, the World Health Organization (WHO) declared the coronavirus disease 2019 (COVID-19) a global health pandemic. COVID-19 is a disease caused by a virus belonging to the large family of coronaviruses. Studies have indicated that the corona virus that causes COVID-19 disease spreads from person to person through small respiratory droplets [1]. The disease has spread worldwide [2]. Various countries have responded differently to the threat of COVID-19. The most common response (though to varying degrees) has been the concept of ‘lockdown’. A lockdown is simply a government-issued emergency protocol that prevents people from leaving a given space. Some governments around the world implemented full lockdowns, while others implemented partial lockdowns [3, 4]. There have been several scholars in support of lockdowns in general. Prominent among them is Neil Ferguson and his team at Imperial College who demonstrated through mathematical models that in the UK alone, a lockdown would save over one hundred thousand lives [5]. These models were crucial in influencing a wave of lockdowns around the world. Other scholars pointed out that lockdowns enabled countries ‘buy time’ in order to get more familiar with the virus to guarantee appropriate responses [6]. The Lancet editorial team also argued that lockdowns were necessary to prevent a collapse of health care systems around the world [7]. Several other scholars expressed similar concerns by highlighting the dangers of increased numbers of new cases which were unmanageable given the limited number of bedspaces, necessary medical equipment and staff in most hospitals around the world [8]. The other popular argument put forward by proponents of a lockdown was the need to ‘flatten the curve’ [4, 9]. This simply meant countering the exponential spread of the virus to keep the prevalence and death rates as low as possible until a possible cure or vaccine was discovered [10].

While lockdowns were embraced and implemented by most countries, the trend also attracted a plethora of critics. Lockdowns were criticized for relying on questionable projections which ended up delaying and worsening the COVID-19 crisis as it prevented the development of herd immunity [11]. Herd immunity occurs when a substantial proportion of the population develops protective antibodies after recovery from the disease [12]. In the absence of a known cure or vaccine, some scholars contended that herd immunity seems like the most plausible alternative. However, given the prolonged lockdowns, thorough development of heard immunity was inhibited [13]. Other scholars point out that lockdowns have less value in containing a virus that has already spread beyond control [14] The argument here is that in a situation where the contagion and the actual number of infections are unknown, blindly locking down countries is merely a symbolic venture that does nothing to stop the spread [10]. Lockdowns have also been criticized for intensifying psychological and socioeconomic situations of citizens. Armitage and Nellums for example showed that COVID-19-related lockdowns led to severe anxiety and depression, especially among the elderly [11]. This is because lockdowns curtail channels of constant social interaction, thereby leading to feelings of loneliness and social isolation which are good breeding ground for anxiety and depression. Critics of lockdowns also believe that despite being well-intended, lockdowns could be producing more harm than good vis-à-vis the socioeconomic conditions of people, especially those living in countries like Zambia which has low income and weak healthcare systems [3]. Specifically, lockdowns are said to have led to high rates of job losses, businesses closures, slowed production process, reduced farming activities and distorted supply chains; all these disruptions are believed to negatively affect the economy and the consequences can be worse for marginalized people [3].

While there is ample amount of research on relevance of lockdowns in responding to the COVID-19 crisis in high income countries, such kind of studies are missing in low-income countries like Zambia [9]. More importantly, it is not yet known how people in such countries view and experience lockdowns. Hence this study aims to fill this gap by investigating the relevance of lockdowns as a tool for fighting COVID-19 by using Lusaka, Zambia as a case study.

Zambia makes a good case study because it was one of the first countries in Sub-Saharan Africa to implement a country-wide lockdown [15]. On the 13th of March 2020, after recording 2 cases of COVID-19, then the president of the republic of Zambia Mr. Edgar Chagwa Lungu issued a statutory instrument number 22 of 2020 on COVID-19. This statutory instrument outlined some of the measures and directives to help combat further spread of the disease [15]. The measures included a partial lockdown of the country in which schools, some shops, restaurants, entertainment activities, religious activities and other forms of social interaction were to be stopped with immediate effect. Citizens were ordered to stay at home as much as possible, and in order to help enforce these measures, police officers were deployed in the streets. Despite the implementation of these measures, it is still not known how citizens view and experience them. Understanding the various ways populations interpret and experience a given health intervention is key to ensuring program effectiveness as health interventions are most effective when they resonate with the worldviews and perceived interests of the people [16]. Lusaka for example which is capital city of Zambia with high numbers of people living in poverty in absolute terms, and has  high rates of Human immunodeficiency virus (HIV) and other tropical illnesses, high density population and the lowest doctor to patient ration poses the greatest challenge [15, 17–20]. Against this backdrop, this study explores the ways in which residents of Lusaka, Zambia characterize the relevance of the lockdown as a tool for fighting COVID-19. Our aim is not to evaluate the impact of the lockdown, but rather to explore, examine and document these local understandings.

## Methods

### Ethical clearance

We obtained a written ethical clearance from (a) The National Health Research Authority of Zambia, and (b) the Zambian Eres-converge ethics board. We also ensured that participants were thoroughly informed of the objectives of the study and of their right to opt out of the study at any point when they feel the need to do so. After agreeing to participate, participants provided us with their written informed consent.

### Study site

The study was conducted in Lusaka, Zambia, the political and economic capital of Zambia. At the time of the study, Lusaka had recorded the most cases of COVID-19 [3]. Lusaka is also the most culturally, ethnically, and religiously diverse city in Zambia. These reasons made Lusaka the best-case study site as it allowed for relevance of the study and also enabled us to have a diverse set of participants in order to enrich our findings.

### Research design

We recruited our participants through convenient and purposive sampling techniques. This was done through the use of the ZAMTEL public phone records. Initial contact was made to potential participants, and they were asked of their availability and willingness to participate in the interview. Upon agreeing to participate, they were included in the sample. A total of 68 people were selected to take part in this study. Their age ranged from 20 to 76 years old. 33 of them were male and 35 were female. In the recruitment process, we also ensured diversity in terms of marital status, ethnicity, employment status, and religious affiliation. Our participants were drawn from various low, middle and income residential arears of Lusaka. Due to COVID-19 restrictions, our interviews were conducted via telephone in conformity with the recommendations from the IRB in Lusaka and the advice of the ministry of health.

### Data collection

We collected our qualitative data by using phone interviews. This was done with the help of 4 local research assistants. The research assistants were seasoned researchers; however, they also received additional training from the researcher responsible for this study in order to enable them to conduct the research ethically and efficiently. The study took place from 13rd to 29th of April 2020. The justification for collecting qualitative data was based on the fact that our aim was to explore lived experiences of Zambian locals regarding the lockdown.

With the guidance of the Social Represenation Theory (SRT), we formulated 13 general questions covering issues regarding experience with the lockdown, opinions on its usefulness and limitations. We also asked follow-up questions to ensure thorough discussions (see interview guide). Each of the interviews took an average of 30–40 minutes. Chinyanja the local language was used during the interviews and English were possible. The interviews were digitally recorded and then later translated and transcribed.

### Data analysis

During the analysis stage, we were guided by the SRT to conduct thematic analysis. Thematic analysis technique is a method of analysis that helps build and organize themes arising from the data. It works by systematically organizing, examining, summarizing and describing emerging themes arising from the data. In this regard, similar opinions presented by our participants were logically clustered to form themes that gave meaning to the data.

## Results

Our participants highlighted the complex interplay of the usefulness of the lockdown as a response to the COVID-19 pandemic as shown in Table 1. All in all, our results are divided into two broad categories, namely, positive and negative characterization of the lockdown.

**Table 1:** Summary of qualitative results

Global theme	Codes
Positive characterization	Contains the spread of COVID-19 Helps prevent the possibility of overwhelming the healthcare system Helps in building a coordinated response Gives government time to prepare appropriate responses It’s the most logical response to a novel contagious disease
Negative characterization	Closes down opportunities for people to make money Leads to job loses Leads to bankruptcy and closure of businesses Increases poverty at household level Inhibits opportunities for the government to raise resources It is an overreaction to a problem that is not as bad as in many other diseases Gives law enforcement agencies legitimacy to be violent towards citizens Worsens mental health Increases rates of domestic violence Increases the risk of child sexual abuse in homes Might give government a basis to scale up and justify oppression Gives raise to government corruption through the lack of citizen engagement There fewer number of deaths does not justify the lockdown

### Positive characterization

On one hand, some participants expressed positive sentiments on the lockdown; they highlighted in many ways how useful the lockdown was. Specifically, the lockdown was credited for containing an impending health disaster. Participants were of the view that an unmitigated approach would have overwhelmed the healthcare system and caused more deaths.

We can already see what is happening in European countries. Look at Italy. They having thousands of people dying every day. Soon it will be us. We don’t have a health care system that can handle the intensity of this virus, so I think the president did well to close down the country. [Lusaka resident,LR21, unemployed]

Participants were also concerned about the possibility of overwhelming the healthcare system in the country. They thus stated that the lockdown was useful in allowing government ‘buy time’ and ensuring that the already-struggling healthcare system was not overrun by several COVID-19 cases. They viewed this as a necessary step to curb the number of deaths that could result both from COVID-19 itself and the lack of capacity to handle other illnesses.

Do you think we have hospitals and ICU spaces to take in all the people who might need care? Should they contract the virus? How prepared are we for this? Look, if they didn’t lockdown, deaths would have piled up because our hospitals are too weak to absorb all the health care needs that would result from high number of cases, and we would see more deaths. [Lusaka resident,LR11 self-employed]

Our participants also observed that given the uncertainties surrounding the novel virus, the lockdown seemed like the most logically-sound response. This action was both cautious and gave authorities time to learn more about how the virus behaved in order for them to design appropriate responses. They argued that it would have been careless to maintain the status quo given the potential consequences of such an action.

Nobody knew how the virus will behave, everybody all over the world was locking down because of the uncertainties surrounding this virus. So to me it makes sense that the first thing you do is lock down than gamble with the lives of people. [Lusaka resident, LR43 employed]

### Negative characterization

While there were sentiments in support of the lockdown, some of our participants remained critical of it. They were concerned about its application and the consequences resulting from it. Particularly, some participants pointed out that the lockdown closed down possibilities of raising income for the most vulnerable. They stated that their lives were dependent on the daily errands which included various economic activities such as vending, hawking, and doing part-time jobs. All these avenues of raising income were closed down and without any corresponding government support, their economic situation became dire.

I survive by vending. I sell tomatoes on the streets, that’s how I raise money to feed myself and my kids. I don’t have savings, this is how I survive. Look at me now, look how hungry we are. You think this is a logical thing to do? To starve us all out in the name of protecting the healthcare system? [Lusaka resident, LR55, unemployed]

Not only that, others complained about the increasing threat of job losses because of the lockdown. Participants highlighted that several people within their circles were losing jobs at a rapid rate. This meant that most families were increasingly finding it difficult to meet their daily needs and poverty at household level was on the rise. They also pointed out that this trend was also detrimental to the government which was losing ground to raise taxes necessary to maintain various social services functional in the country.

Companies are closing down and people are being let go. Unemployment is on the raise in the townships. People are struggling. Now, if companies are closing down, where will government find the money to fund health care, education, electricity? Where? [Lusaka resident, LR08 retired]

The lockdown was also accused of contributing to the deterioration of mental health in the country owing to reduced human social interactions. Participants highlighted that Zambian societies were based on communal connections which people relied on for various forms of support. Cutting this off meant that people were left feeling isolated, stressed out and lonely.

I have never felt like this before. I am not allowed to see my friends. I am not allowed to go to the bar and have a beer, talk about my problems to my friends, and for help where I can. I just feel so stressed locked down in this small space. These people are killing us with stress and loneliness. [Lusaka resident, LR 16 unemployed]

Participants also complained of the increased number of gender-based violence and sexual assault in their circles. They stated that the lockdown had caused a disruption in normal day-to-day lifestyle activities in society and thus family members were meant to stick together more than usual. This had the potential of causing resentment towards each other thus leading to more women becoming victims of violence at the hands of their male counterparts. Vulnerable children were also said to be at higher risk of sexual assault given the constant presence of male family members in close proximate.

Now we are hearing more of violence against women in the neighborhood. We are getting reports of excessing violence towards women. It's in the news every day. …This is not good. Also, look at our young girls, you think they are safe in these homes with their uncles, cousins, brothers. You know the stories. What is happening is really sad. [Lusaka resident, LR 44 employed]

The lockdown was also seen as an excuse for government to increase its repression against citizens especially those with dissenting voices. The city of Lusaka was heavily militarized with security officers using excessive force on citizens who were deemed to have broken the lockdown rules. While the ruling party was allowed to have gatherings, other political parties and civil societies were met with excessive police brutality if the dared to do the same. Several other pronouncements were made to intimidate dissenting voices on the pretext of maintaining the lockdown rules. Minus any citizen oversight on the state resulting from the lockdown, abuse of resources by officials was said to be rampart particularly resources donated to the fight against COVID-19.

This is just an excuse for them to silence the opposition and loot our resources. Look at how they beat up and arrested those guys last Friday night. All this militarization of our neighborhoods, men with guns patrolling our neighborhoods, for what? We know these people are stealing all the donations for the COVID-19 fight. It is all over the news and people are not even allowed to ask questions. [Lusaka resident, LR 61 employed]

Further, Zambia had recorded a significantly low number of deaths as compared to other countries. By end of April 2020, the COVID-19-related death count stood at 4. As such, most participants felt the virulence in Zambia did not justify the lockdown. They pointed out that deaths from pandemics were not new in Zambia as the country had previously suffered various contentious pandemics, most of which claimed several thousands of lives in a short period of time but were managed without lockdowns. The consequences of the lockdown when weighed against the death rate at the time seemed counterproductive.

In the last 3 months since Corona started making headlines, the country has only seen 4 deaths, only 4. Cholera kills more people and it is equally contentious. So given 4 deaths and compared to the damage the lockdown is causing, I really think this action is somehow stupid. The lockdown is killing us, not the virus. [Lusaka resident, LR 35 employed]

## Discussion

Our analysis illustrates various ways in which the lockdown was characterized in Lusaka, Zambia. Broadly, the lockdown was understood in terms of its positive and negative consequences in the process of responding to the COVID-19 crisis.

There was evident support of the lockdown from our participants who argued that it was a necessary response in order to slow down the spread of the corona virus, prevent fatalities, and prevent the healthcare system from collapsing. Participants also suggested that failure to take any such measures against a novel virus whose actual infectiousness and virulence was not yet known amounted to recklessness. There were similar support and sentiments from other parts of Africa [5]. The logic behind this position is since Zambia’s health care system had various shortcomings which could be seen from its consistent failure to effectively handle a plethora of illness such as malaria, malnutrition, diarrhea, cancer and HIV/AIDS [18]. Therefore, a further COVID-19-precipitated increase in patient-number without any expansion in capacity of the healthcare system risked collapsing it [2, 21–23]. COVID-19 response requires adequate human resource, availability of Personal Protective Equipment (PPE), ventilators and a constant supply of electricity, all of which were already in short-supply in Zambia [18]. There were several catastrophic predictions of deaths estimated in the range of one hundred to three hundred thousand in African countries like Zambia with high HIV prevalence rates [17]. It thus seemed careless to continue with the status quo. Our participants expressed little confidence in the healthcare systems’ ability to handle an increased wave of COVID-19 cases. As such, a lockdown was seen as a cheaper, more effective and logical means of reducing this risk in the sense that it limits social contact and thereby cuts down on the infectiousness of the virus. Prevention of the virus through lookdowns rather than gambling with people’s lives via a Laissez-faire approach seemed more logical.

While others saw the relevance of lockdowns, there was overwhelming criticism directed towards it [4, 24, 25]. The lockdown was characterized as a disproportionate, out-of-touch and drastic response that ignored its own resulting consequences which were in most cases worse than the problem it aimed to solve [3, 26–28]. The lockdown was accused of closing down important economic avenues of survival among the most vulnerable in Zambia. A country in which the majority of the people are concentrated in informal economic activities such as street-vending, hawking and manual labor; lockdowns threatened their economic survival. Most families in Lusaka, Zambia survive on ‘hand-to-mouth’, and usually lack savings and the ability to stock up. This means that closing down economic activities without any subsequent government subsidies worsens the already precarious poverty situations of many households. Our participants complained of the economic difficulties they had to endure at the hands of the lockdown whose purpose they did not fully grasp. Several scholars have made similar observations in other African countries. Many of our participants viewed the threat of dying from poverty a more poignant reality than the lottery of contracting corona virus [29].

Other challenges such as increased mental distress, gender-based violence and sexual violence against young girls at home were significantly pointed out. Mental health challenges especially for elderly people without possibilities of social interaction seemed to have been affecting many parts of Lusaka. Other studies have also shown similar trends in countries that have low income and high concentration of populations living in slums [29]. Further, riding on a culture that objectifies and victimizes women, lockdowns became convenient breeding ground for further abuse in Zambia. Participants highlighted their fear and concern on the continued threat of sexual and physical violence towards women in what they called ‘caged-environments’ which provided little opportunities of escape. Similar observations have been made elsewhere in countries like Kenya it was estimated that there was a significant rise in gender-based violence in the country owing to lockdowns [30–34]. Research has shown that lockdowns exacerbate pre-existing gender inequities, power-hierarchies, economic-stressors; as such, they contribute to increasing tension in households. This, coupled with the fact the women are constantly in close-proximate with their (potential) abusers, the frequency and severity of the abuse increases [3].

Our study also demonstrates how the lockdown was used as a convenient excuse for continued political repression, abuse of authority and corruption. Our participants made several observations of how freedoms of citizens especially those with dissenting views were constantly curtailed in the pretext of upholding the norms of the lockdown. The lockdown as suctioned through statutory instrument number 22 of 2020 took away citizens’ ability to provide oversight on the government’s conduct. The president and those in authority were accused of abusing their powers and further normalizing military-sanctioned violence on citizens. Similar reports of state-sanctioned violence on citizens in the name of upholding the lockdown have been reported in other African countries, particularly, Kenya, Uganda and South Africa [26, 28, 32]. Several researchers have raised concerns over the continued breakdown in the rule of law in most African countries hidden under the gaze of promoting public good through lockdowns. Some countries have already reported deaths of citizens at the hands of military personnel in the process of enforcing lockdown measures.

Our participants also questioned the justification of such a lockdown especially when weighed against the number of fatalities resulting from COVID-19. Many suggested that a lockdown (given its consequences) was a disproportionate response to a pandemic that had claimed only 4 deaths in 3 months at the time. They also observed that pandemics in Zambia were not new. The key to Zambia’s resilience against previous pandemics had been the use of mechanisms of response that carefully balanced risk of fatality and socioeconomic stability of the country; in the case of COVID-19, this seemed to have been ignored [9]. As has been established in other low-income countries, the psychological, socioeconomic, governance and political impact of a lockdown may be much greater than the risk posed by the corona virus itself [7]. Against the evidence of various negative consequences resulting from a lockdown, a plethora of scholars have concluded that in low-income countries, it is a counterproductive and disproportionate response [2, 22, 35, 36].

While in some high-income countries the risks identified in this study can easily be managed due to provision of social security benefits, such opportunities may not exist in low-income countries [22, 23, 37]. This means lockdowns may be causing more harm than good in low-income countries as compared to high-ncome countries. The unique realities of low-income countries may be at odds with lockdowns [9].

Overall, our findings suggest a complex and multifaceted characterization of the lockdown. On one hand, it was seen as useful in slowing down the spread of the corona virus, preventing fatalities, and protecting the healthcare system from the risk of collapse. On the other hand, it compounded the predicaments of many households in various ways. Its negative consequences were especially observed in increased poverty levels, unemployment, cutting down lines of economic survival, high mental health problems, increased gender-based violence, high rates of sexual violence, and intensified political repression and corruption. Thus, rather than definitely establishing lockdown as an incontestable good, as it is depicted by some scholars or as useless by its critics. Our findings instead demonstrate the diversity and complexity in how it is locally viewed by Zambians in Lusaka.

Based on our findings, it seems plausible to call for a more complex and context-specific response that tries to provide protection to various risks not only to the risk of COVID-19. This sort of response must endeavor to minimize costs and maximize benefits while galvanizing local buy-in. This would also involve paying attention to the socioeconomic realities and the vulnerabilities of specific demographics due to existing comorbidities and other risks factors such as age. The threats of poverty, sexual assault, unemployment and mental health should be included in any COVID-19 response in Lusaka, Zambia. A more useful response would be one that tackles both COVID-19 as well as risks highlighted in this study. Ultimately, we posit that there cannot be a one-size-fits-all solution or a magic bullet to this predicament, thus our emphasis here is the need for contextualization and complexity in responding to COVID-19.

The above notwithstanding, this study has some limitations. Due to various barriers such as funds, time and lockdown measures, we conducted our interviews by phone and the interviews were limited to Lusaka city alone. We were unable to conduct a country-wide study in order to have more representative responses. However, despite all that, our findings provide novel insights on how the corona-precipitate lockdown is characterized by the people in low-income countries like Zambia.

## Conclusion

With the help of the SRT, we set out to explore and document the local characterization of the lockdown by residents of Lusaka, Zambia. The SRT helped us unpack the various and complex ways the lockdown was viewed in Lusaka, Zambia. Specifically, it was established that the lockdown is on one hand lauded for slowing down COVID-19 incidence rates, preventing fatalities, and protecting the healthcare system from collapse. On the other hand, it is criticized for exacerbating poverty levels, unemployment rates, cutting down lines of economic survival, increasing the rate of mental health problems, aiding gender-based violence, and intensifying political repression and corruption. These results speak to the complexity of a lockdown as a universal response to such pandemics. Thus, the observation demonstrates the folly of viewing, applying and characterizing the COVID-19 lockdown as a ‘one-size-fits-all’ intervention in Lusaka, Zambia. The above notwithstanding, much research remains to be done in order to generalize our findings to other low-income countries. However, we argue that this study provides grounds for caution on simplistic and binary characterization of lockdowns. The study indicates the need for careful dialogue between the designers of lockdowns and citizens in order to tailor such interventions to local realities in context-specific ways. It also shows that through the development of such interventions, all the various and complex elements it embodies must be taken into account in order to realize optimum outcomes.

## Data Availability

The data generated and/or analyzed during the current study will be made fully available to the public.

## Abbreviations

COVID-19: Coronavirus disease 2019
HIV: Human immunodeficiency virus
PPE: Personal Protective Equipment
SSA: Sub-Saharan Africa
STR: Social Representation Theory
WHO: World Health Organization
